# Editorial for “The Role of Ketogenic Diet in Human Health and Diseases”: The Multifaceted Impact of Ketogenic Diets on Health and Disease

**DOI:** 10.3390/nu15184027

**Published:** 2023-09-17

**Authors:** Mikiko Watanabe, Silvia Savastano, Carla Lubrano, Giovanni Spera

**Affiliations:** 1 Department of Experimental Medicine, Section of Medical Pathophysiology, Food Science and Endocrinology, Sapienza University of Rome, 00161 Rome, Italy; carla.lubrano@uniroma1.it (C.L.); giannispera@yahoo.it (G.S.); 2 Endocrinology Unit, Department of Clinical Medicine and Surgery, University of Naples “Federico II”, 80131 Naples, Italy; sisavast@unina.it

The ketogenic diet (KD), characterized by a very low carbohydrate intake and variable protein, fat and calorie intake, has long been in the spotlight for its potential therapeutic applications. Only a short time ago, the KD was seen as a fad diet [1], but since then, many studies have been conducted, confirming its efficacy and safety. As ketone bodies seem to have a multidimensional role, several applications are now being proposed even beyond simple metabolic effects, most importantly leveraging their immunomodulating impact. Our Special Issue has brought together an array of studies that delve into the diet’s effects on various aspects of metabolic and endocrine health and disease, ranging from its impact on insulin secretion and liver health to its role in managing specific conditions like Lennox–Gastaut syndrome and inflammatory diseases.

The metabolic benefits of KD are among its most well-documented effects. In this regard, an intriguing finding comes from the study by Battezzati et al., which investigated the acute insulin secretory effects of a classic ketogenic meal in healthy subjects. The study found that a ketogenic meal elicited only a minimal insulin secretory response compared to a Mediterranean meal [2]. This is particularly relevant for patients with insulin resistance or insulin secretory defects, as it suggests that a KD could potentially mitigate hyperinsulinemia and improve insulin sensitivity. Studies by Rinaldi et al. and Cincione et al. further extend the diet’s benefits to liver health and obesity management. The former showed significant improvements in non-alcoholic fatty liver disease (NAFLD) parameters [3], while the latter demonstrated improvements in patients with prediabetes or diabetes and overweight/obesity [4]. Zahra Ilyas et al. aimed to define the effectiveness of KD for the management of sarcopenic obesity, supporting the evidence that KD improves metabolic health and body composition [5]. Metabolic and cardiovascular disease are intertwined, and Sánchez et al. explored the diet’s effects on adventitial vasa vasorum density, a biomarker of early atheromatous disease. While the density did not change significantly, the study found improvements in markers of endothelial dysfunction and cardiovascular risk [6]. This suggests that while the diet may not directly impact certain biomarkers, it could still offer cardiovascular benefits through other mechanisms. These studies propose the KD as a viable strategy for managing liver health and other metabolic disturbances, confirming the findings of previous studies.

The mechanisms via which nutritional ketosis is capable of improving metabolic health are far from being completely elucidated [7,8,9]. However, some are relatively well established, and some are currently being studied. For example, Rossella Tozzi and colleagues provided a narrative review on ketone bodies and SIRT1 as synergic epigenetic regulators for metabolic health. The review offered insights into their central roles in metabolic health [10]. Alessio Basolo et al. explored the influence of KD on energy expenditure in the context of weight loss, discussing the dynamic changes in energy expenditure during different phases of KD [11]. Immunomodulation is surely a key mechanism of action of ketone bodies [12,13], making this approach potentially viable for several conditions where low-grade inflammation is pathogenetic [8]. For example, studies examining the effects of KD on male accessory gland inflammation offer a glimpse into the diet’s broader applicability in urological conditions. While the mechanisms are not yet fully understood, the diet’s anti-inflammatory and metabolic effects are likely contributing factors [14].

The neuroprotective effects of KDs are another area where this dietary approach shows significant promise. The article by Bölsterli et al. opens a new avenue in the treatment of mitochondrial malate aspartate shuttle system and mitochondrial pyruvate carrier deficiencies, which are associated with neurological phenotypes. The study shows that KDs can be beneficial, particularly against seizures, in patients with these defects [15]. This adds to the growing body of evidence supporting the use of KDs in various neurological conditions, including Lennox–Gastaut syndrome, a form of epilepsy, as discussed by Skrobas et al., who suggest that ketogenic dietary therapies can provide significant seizure reduction and are generally safe when applied under medical supervision [16].

The potential role of KD in cancer therapy is an exciting and rapidly evolving area of research. Simone Dal Bello and associates reviewed the potential of KD in the treatment of gliomas and glioblastomas. The review emphasized the need for further studies to validate the role of KD in cancer therapy [17]. More broadly, Noushin Mohammadifard and team reviewed the effect of KDs on shared risk factors of cardiovascular disease and cancer, discussing underlying potential mechanisms of action [18].

Several new strands of research are also emerging. The relationship between KD and gut microbiota is a novel field of study that has already shown promising results. Research has indicated that KD can significantly alter the composition of gut microbiota, leading to increased production of beneficial short-chain fatty acids like butyrate [19]. Alsharairi’s review focused on the diet’s potential to modulate infant gut microbiota via nutritional changes in the mother during pregnancy and lactation. Microbiota alterations may induce epigenetic changes in genes related to obesity and modulate inflammation in adipose tissue [20]. Another emerging field is the diet’s influence on hormonal and circadian rhythms. Masi et al. reviewed the literature on how ketogenic diets affect processes that follow circadian rhythms, such as appetite and sleep [21]. This opens up an exciting avenue for future research into how the diet could be timed to optimize its benefits.

While the KD holds promise, it is not without its controversies and limitations. Concerns have been raised about the diet’s long-term safety, particularly regarding lipid profiles and cardiovascular health [22]. Moreover, type 1 diabetes (T1D) was traditionally considered as an absolute contraindication to the KD [23]. Andrea Kleiner and team evaluated the safety and efficacy of a eucaloric very-low-carb diet in T1D, reporting improved glycemic control and reduced insulin requirements, supporting the diet’s safety and effectiveness under medical supervision in the context of this disease [24]. This confirms that the landscape is rapidly evolving regarding new applications, potential safety concerns, and mechanistic explanations.

As we move toward an era of personalized medicine, the KD offers a promising avenue for individualized treatment plans. Ongoing research is exploring the genetic and epigenetic factors that may influence an individual’s response to the diet, as well as the potential for using biomarkers to predict efficacy and safety. These advances could pave the way for more targeted and effective use of the ketogenic diet in clinical practice. Our Special Issue serves as a testament to the growing interest and accumulating evidence in favor of KDs as a versatile therapeutic option, offering a glimpse into their therapeutic potential that is as promising as it is diverse. As we continue to delve into the complexities of this diet, it becomes increasingly clear that its full therapeutic potential is yet to be unlocked. While the KD is not a one-size-fits-all solution, its potential applications are broad and warrant further investigation. It is an exciting time for KD research, and the coming years are likely to bring even more insights into its myriad applications and mechanisms of action, but rigorous, multidisciplinary research is essential to fully understand and harness the transformative power of the KD in modern medicine.
